# The neuroimmune-neuroplasticity interface and brain pathology

**DOI:** 10.3389/fncel.2014.00419

**Published:** 2014-12-04

**Authors:** Shawn Hayley

**Affiliations:** Hayley Lab, Neuroscience, Carleton UniversityOttawa, ON, Canada

**Keywords:** neuroplasticity, cytokine, neurodegeneration, inflammatory, BDNF, depression, neurogenesis, erythropoietin

It is now well-established that the brain, although immune privileged, has fundamental interactions with elements of the immune system. Perhaps, one of the most exciting aspects of such neuro-immune communication, has been the notion that peripheral derived immune factors (e.g., lymphocytes and cytokines), as well as central glia (particularly microglia) are thought to participate in the modulation of a variety of psychiatric and neurological conditions. At the very least, microglial and cytokine responses are though to shape some aspects of disease progression and possibly the emergence of comorbid features. One basic mechanism through which immune cells (including microglia) and soluble immunotransmitters could influence a variety of brain conditions is via changes in neuroplasticity.

Virtually all psychiatric (e.g., major depression) and neurological [e.g., Parkinson's (PD) and Alzheimer's disease (AD)] disorders have been associated with changes in neuroplasticity and neuroinflammatory processes (Frank-Cannon et al., 2009; Hayley, 2011). Indeed, reductions of adult hippocampal neurogenesis, diminished cortical dendritic arbors, deficits in long-term potentiation (LTP) and impaired synaptogenesis have all been reported (Perederiy and Westbrook, 2013; Na et al., 2014). Papers in the present special topic discuss evidence that the pro-inflammatory factors [e.g., interleukin-1β (IL-1β), tumor necrosis factor-α (TNF-α)] provoke neuroplastic deficits, whereas anti-inflammatory and neurotrophic cytokines enhance neuroplasticity and might even have neuroprotective or regenerative effects (e.g., Hayley and Litteljohn, 2013).

This Frontiers Research Topic is comprised of a series of articles that deal with how brain-immune system interactions occur and their influence on neuroplasticity. Several interrelated key issues were touched upon, namely: (1) The specific neuroinflammatory pathways that contribute to neurodegeneration and how targeting these pathways might facilitate recovery by promoting neuroplasticity, (2) The role of pro-inflammatory cytokines and immune cells in the cognitive and behavioral deficits associated with head injury or neurological illness, (3) How inflammatory environmental stressors promote deficits in neuroplasticity and behavioral functioning, and (4) How signaling molecules of the immune system, particularly trophic cytokines, might be utilized as novel antidepressant or neurorecovery agents.

While the housekeeping functions of central and peripheral inflammatory immune cells are normally beneficial and buffer neurons against deleterious insults, the over-activation of neuroinflammatory cascades is exceedingly dangerous to neurons. For instance, in the case of PD, as pointed out in the article by Pitossi's group in this special issue, accumulating data support a link between PD and enhanced microglial and pro-inflammatory cytokine (e.g., TNF-α and IL-1β) responses (Leal et al., 2013). Similarly, Rivest and colleagues provide compelling evidence to suggest that key components of the IL-1 signaling system, namely IL-1RAcPb, influence the long-term survival of neurons exposed to an excitotoxic insult (Gosselin et al., 2013). Understanding exactly why pro-inflammatory factors become dysregulated in the first place has been an enormous challenge and likely reflects a combination of events, including genetic mutations in “plasticity” and “inflammatory” genes [e.g., Brain-derived neurotrophic factor (BDNF) Val66Met polymorphism and Leucine Rich Repeat Kinase (LRRK2), respectively], coupled with exposure to environmental insults.

Developing means of enhancing the endogenous repair mechanisms represents one viable approach to battling mental health and neurological conditions. In this regard, taking advantage of the natural capacity of immune factors to exhibit “learned” responses and to traffic to and interact with cells at sites of pathology may be of particular importance. Hayley and Litteljohn (2013), in this issue, discuss the cytokine, erythropoietin (EPO), with respect to its potential antidepressant effects. Indeed, EPO is considered as one of a potential next wave of agents with antidepressant properties posited to stem from their ability to modulate aberrant neuroplasticity in emotion regulatory brain circuits. Conversely, cytokines with primarily pro-inflammatory properties, such as TNF-α and IL-1β, as outlined in the Audet and Anisman (2013) paper, have been reported to adversely affect neuroplasticity and promote depressive illness. Mallimo and Kusnecov (2013) go further still to illustrate how neuroinflammatory cytokines affect behavioral outcomes through their impact on neuropeptide signaling pathways. Ultimately, this line of investigation could identify novel targets for treating stressor related disorders.

Besides symptom management, it is of particular importance to develop strategies that address the underlying pathology and either reverse/stabilize neuronal derangements or alternatively, promote compensatory recovery of existing circuits. In this regard, Munemasa and Kitaoka (2013), in this issue, report intriguing findings indicating a role for TNF-α in retinal cell degeneration in glaucoma and raise the possibility that modulation of BDNF might not only have neuroprotective functions, but also facilitate axonal regeneration. Interestingly, the article by Holahan's group also highlights the role of pro-inflammatory factors in cerebral concussion and head injury (Patterson and Holahan, 2012). This is a case where it is critical to assess the time-dependent emergence of neurological sequelae that might coincide with the progression of inflammatory cascades. Thus, anti-inflammatory treatments might have prophylactic utility in such cases and identification of inflammatory biomarkers of disease progression might help in “fine tuning” the treatment strategy adopted for individual cases.

The intersection between genetic and pharmacological based approaches for brain conditions is a particularly important area. As reported by Liu et al. (2014) in this issue, the use of induced pluripotent stem cell (iPSC) technology allows one to tailor specific cell replacement approaches using an individual's own somatic cells. In addition to potentially replacing loss neurons in cases of neurodegeneration, the iPSC approach might be useful in the remediation of aberrant structural changes through the enhanced long-term expression of BDNF or other trophic factors. Indeed, central infusion of iPSC-derived neural cells that co-expressed BDNF increased subventricular zone neurogenesis and blunted the corticoid response to a stressor (Liu et al., 2014).

Finally, Rivest and colleagues have been at the forefront of examining neuroimmune interactions and brain plasticity and in this issue they provide captivating evidence to suggest that defects in monocyte subsets contribute to cognitive dysfunction in AD. Importantly, they show that administration of the trophic cytokine, macrophage-colony stimulating factor, reversed the cognitive decline and hematopoietic deficits observed in a transgenic model of AD (Naert and Rivest, 2012).

Ultimately, it is important to consider both the protective and destructive aspects of the inflammatory immune system and how the microenvironment in which these molecules act is critical. Indeed, beneficial vs. deleterious effects can even vary over time with any particular illness. As pointed out by Patterson and Holahan, this might explain the lack of positive clinical effects for general use of broad-spectrum anti-inflammatory drugs (e.g., NSAIDs) in the treatment of brain pathologies. In effect, the path forward should involve drugs aimed at targeting specific inflammatory factors (e.g., TNF-α, IL-1β, IFN-γ) identified by several laboratories, including those of Anisman et al., depending upon the particular condition and stage of disease. That said, boosting protective aspects of immunity, as reported by papers by each of Rivest et al., as well as Hayley, suggest that interventions that elevate endogenous trophic cytokines could have immense clinical benefits.

## Conflict of interest statement

The author declares that the research was conducted in the absence of any commercial or financial relationships that could be construed as a potential conflict of interest.
